# Observations Regarding the Detection of Abnormal Findings Following a Cancer Screening Whole‐Body MRI in Asymptomatic Subjects: The Psychological Consequences and the Role of Personality Traits Over Time

**DOI:** 10.1002/jmri.29461

**Published:** 2024-05-31

**Authors:** Lorenzo Conti, Davide Mazzoni, Chiara Marzorati, Roberto Grasso, Derna Busacchio, Giuseppe Petralia, Gabriella Pravettoni

**Affiliations:** ^1^ Applied Research Division for Cognitive and Psychological Science European Institute of Oncology IRCCS Milan Italy; ^2^ Department of Oncology and Haemato‐Oncology University of Milan Milan Italy; ^3^ Division of Radiology IEO European Institute of Oncology IRCCS Milan Italy

**Keywords:** whole‐body MRI, cancer screening, psychological consequences, psychosocial health, abnormal findings

## Abstract

**Background:**

The use of whole‐body MRI (WB‐MRI) in oncology has uncovered frequent unexpected abnormal findings (AFs). However, the impact of AFs on the patients' mental well‐being is still poorly examined.

**Purpose:**

To investigate the long‐term psychological consequences of AF detection following WB‐MRI for cancer screening in asymptomatic individuals.

**Study Type:**

Prospective, longitudinal.

**Population:**

121 consecutive subjects of the general population (mean age = 52.61 ± 11.39 years; 63% males) scheduled for cancer screening by WB‐MRI.

**Field Strength/Sequence:**

1.5‐T and 3‐T; protocol complied with Oncologically Relevant Findings Reporting and Data System (ONCO‐RADS) guidelines.

**Assessment:**

Participants completed the first psychological investigation (T0) immediately after the WB‐MRI. Subsequently, it was repeated after 1‐year (T1), and 4‐years (T2, subgroup of 61 participants) without an MRI exam, assessing personality traits, tumor risk perception, quality of life, depressive, and anxious symptoms. Radiologists directly reported WB‐MRI findings to the participants, explaining the clinical implications and the location of the AFs. The number and severity of AFs were assessed.

**Statistical Tests:**

Pearson's correlations and analysis of variance with repeated measures assessed the psychological health variables' relationship and their changes over time. A *P*‐value <0.05 was considered statistically significant.

**Results:**

All participants presented AFs, with 101 individuals categorized as ONCO‐RADS 2 and 19 as ONCO‐RADS 3. The AFs were most prevalent in bones (31.5%). The overall participants showed only a slight increase in depressive symptoms at T1 [*F*(1,112) = 7.54]. The severity and the number of AFs were not significantly related to psychological changes [ranging from *P* = 0.503 to *P* = 0.997]. Depressive and anxious symptoms over time were significantly affected by the traits of conscientiousness [T1: *F*(1,112) = 7.87; T2: *F*(1.708,90.544) = 3.40] and openness [T1: *F*(1,112) = 4.41].

**Data Conclusion:**

Disclosing AFs by WB‐MRI exams for cancer screening may not lead to long‐term psychosocial consequences. Certain personality traits may, however, influence the psychological distress experienced by individuals with AFs after WB‐MRI exams.

**Level of Evidence:**

2.

**Technical Efficacy:**

Stage 5.

Whole‐body MRI (WB‐MRI) is a radiation‐free imaging method for detecting bone and soft tissue pathology that is playing a growing role, thanks to the multiple applications in disease detection.^1^, ^2^ In particular, the excellent diagnostic performance of WB‐MRI in tumor detection (i.e., overall sensitivity of 90%) has expanded its application in oncology to the point of being recommended in international guidelines for the management and assessment of several typologies of cancer.^2^ These advantages, together with the lack of radiation exposure and absence of contrast‐agent administration in a typical WB‐MRI examination, have contributed to increasing attractiveness for cancer screening as an adjunct to traditional cancer screening examinations (eg, mammography, pap‐test, fecal occult blood testing, and prostate‐specific antigen evaluations). Despite the growing relevance and promotion of cancer screening due to the clinical benefits for patients, several factors are still hindering the introduction of WB‐MRI within routine clinical practice. The high cost of the scanner and the requirement of good clinical expertise in WB‐MRI interpretation have limited its availability nationwide. Only a few specialized medical centers have WB‐MRI scanners representing a major barrier restricting access to care for the general population, especially those living in rural areas. However, literature has shown that the adoption of WB‐MRI in cancer surveillance programs is cost‐effective for the early detection of cancer and the staging of metastatic disease compared with conventional imaging.^3^ These advantages have extended the adoption of WB‐MRI to early cancer detection in subjects with cancer predisposition syndromes and the general population.^1^, ^4^ Specifically, preventive exams may lead to detection in the earliest stages of cancer, allowing precision healthcare treatments with appropriately targeted interventions, which could drastically modify disease development and improve survival rates.^5^

Several studies examined the adoption of WB‐MRI for cancer screening in the general population, focusing on the prevalence of relevant, and indeterminate findings.^6^, ^7^, ^8^ Indeed, specific guidelines such as Oncologically Relevant Findings Reporting and Data System (ONCO‐RADS) allow us to determine if the outcome of the WB‐MRI exam is normal or not, and to assign the likelihood of malignancy of abnormal findings (AFs) by using a five‐point category assessment score.^9^ Specifically, AFs can be highly likely to be benign (ONCO‐RADS 2), likely to be benign (ONCO‐RADS 3), likely malignant (ONCO‐RADS 4), or highly likely to be malignant (ONCO‐RADS 5; eg, histological examination and/or immediate clinical classification required).^9^ In this context, WB‐MRI leads to the detection of a substantial number of AFs, with potential health consequences for the subjects.^6^, ^10^ A literature review revealed that AFs are expected in about 95% of asymptomatic subjects undergoing WB‐MRI for cancer screening, but <2% would be reported as suspicious for malignant diseases.^6^ This implies that most of them do not imply health consequences for the patients.

Given the high occurrence of AFs in the general population, the investigation of the impact that their discovery on individual quality of life (QoL) and emotional states may be crucial for implementing screening programs and improving the patient's experience. In this regard, a previous study conducted on cancer patients showed psychological distress related to the outcome of the examination immediately after the screening, thus highlighting the important relationship between the patient's emotional state and the clinical report. Moreover, individual characteristics have been shown to affect participant's perspectives regarding the WB‐MRI exam: other studies highlighted a positive correlation between personality traits and satisfaction with the WB‐MRI procedure.^11^, ^12^, ^13^ Only a few studies focused on the psychological correlates of the disclosure of AFs in asymptomatic individuals.^14^, ^15^, ^16^ More specifically, one study examined 394 volunteers, showing that the disclosure of AFs following WB‐MRI induced short‐term effects with moderate to severe psychological distress.^15^ A difference was also found between the patient's subjective interpretation and radiological assessments of the severity of AFs. It was communicated indirectly to the subject, promoting misinterpretation of the health outcome and subsequent discomfort. Conversely, other studies^14^, ^16^ explored the long‐term psychological impact of WB‐MRI in a sample of subjects of the general population: Schmidt et al found that patients with AFs according to WB‐MRI reported no relevant effects on either QoL or depressive symptoms after ~2–3 years.^16^ Accordingly, in a recent study, Korbmacher‐Böttcher et al reported that individuals who voluntarily participated in WB‐MRI studies had less psychosocial burden compared with the control group, and the AFs reported were not associated with adverse long‐term psychosocial consequences.^14^

Psychosocial aspects such as anxiety and depressive symptoms and their impact on QoL have been investigated in the previously mentioned studies,^14^, ^15^, ^16^ but numerous other features may contribute to the subjects' interpretation of health outcomes. Indeed, both individuals' perception of an event and personality characteristics influenced behavioral responses to health news.^17^, ^18^, ^19^ However, many of these aspects have not been sufficiently investigated in subjects who underwent WB‐MRI.

Against this background, the objective of this study was to evaluate the long‐term impact of AFs disclosure on asymptomatic participants of the general population, who underwent a WB‐MRI examination for cancer screening. Furthermore, we aimed to assess whether AFs may affect participants' psychological health outcomes and how individual personality and risk perception could affect the AFs interpretation.

## Material and Methods

This prospective study was approved by the ethical committee of the Scientific Institute for Research, Hospitalization and Healthcare (IRCCS) European Institute of Oncology (1032_UID_1810). Written informed consent was obtained from all subjects involved in the study. The present research study followed the Strengthening the Reporting of Observational Studies in Epidemiology (STROBE) statement designed for cohort studies (See Data S1).

### Participant Sample

This study considered asymptomatic subjects who underwent WB‐MRI examinations for cancer screening either as part of a check‐up program at the IRCCS European Institute of Oncology or as a stand‐alone WB‐MRI based on external information (eg, internet, word of mouth). The subjects had independently decided to undergo the WB‐MRI exams as an adjunct to standard screening examinations.

Individuals who had booked a WB‐MRI exam were contacted by telephone or email to propose participation in the study. Those who showed interest were scheduled for an interview at the European Institute of Oncology on the day the WB‐MRI was performed to fill in the informed consent.

Participants included had no psychiatric condition or other disorders that could have prevented the participation in the study and had no contraindications for MRI examinations (eg, pacemaker, pregnancy in the first trimester, and metal implants).

**FIGURE 1:**
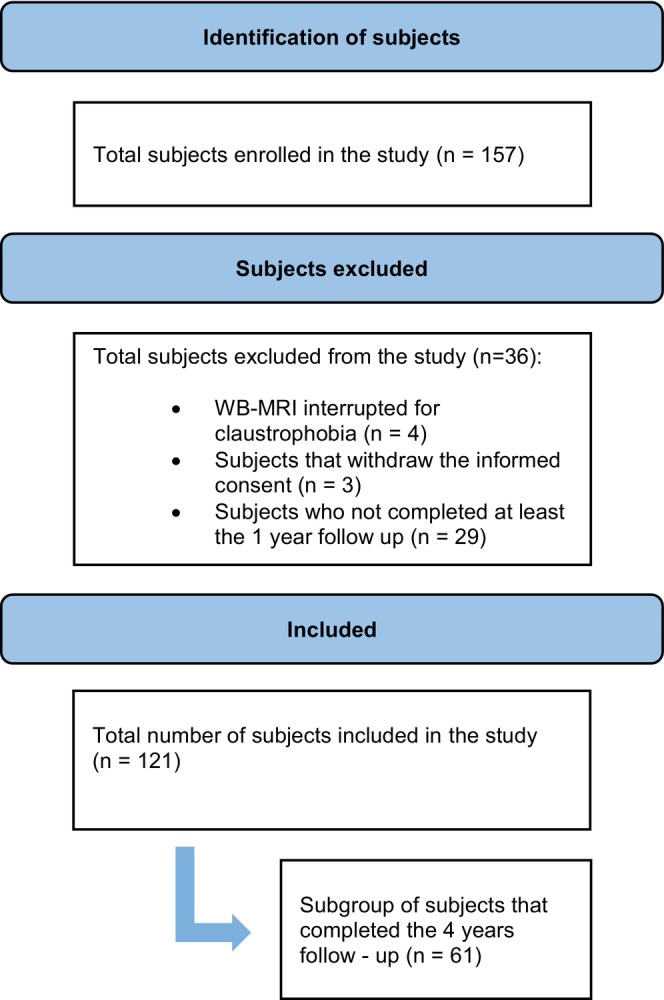
Flow chart of the study population and data availability.

One hundred and fifty‐seven asymptomatic subjects participated in this prospective observational study between July 2019 and July 2023. After the exclusion of 36 subjects due to several reasons (Fig. 1), 121 subjects were included in this study. All these subjects underwent a psychological assessment and a WB‐MRI on the same day. The psychological assessment was performed again after 1 year, without an additional WB‐MRI. After 4 years, only a subgroup of subjects accepted to be recontacted for a long‐term examination, reperforming the psychological assessment (see Table 1).

**TABLE 1:** Timing of Assessment*Only a Subgroup of Subjects (*N* = 61) Performed the T2 Assessment

Data Collected	Timing of Assessment		
	T0	T1	T2*
Demographycal variables (age, gender, ethnicity, and educational level)	X	–	–
Personality traits	X	–	–
Risk perception	X	–	–
Psychological health variables (anxiety, depression, and QoL)	X	X	X
WB‐MRI examination	X	–	–
Acceptance of the WB‐MRI	X	–	–

### WB‐MRI Procedure

All participants were first informed about the WB‐MRI procedure (eg, scanning duration and behavior to follow during the exam).

The WB‐MRI protocol was compliant with the ONCO‐RADS guidelines,^9^ including axial spin‐echo echo‐planar diffusion‐weighted, gradient echo T1‐weighted dual‐echo Dixon, and half acquisition turbo spin echo T2‐weighted images from the skull base to mid‐thigh, sagittal fat saturated T2‐weighted images of the spine, axial fluid‐attenuated inversion recovery T2‐weighted image of the brain and axial short echo time T1 of the lungs, with a duration of about 40 minutes (see Data S1 for details). Two scanners were used for this study (1.5 Magnetom Avanto or 3 T Magnetom Skyra, Siemens Healthineers, Erlangen, Germany). Anatomy‐specific phased‐array surface coils were used for all body regions.

### Psychological and Clinical Assessment

All participants, after signing the informed consent and before the WB‐MRI exam, met the psychologists with 6 and 4 years of expertise in clinical psychology for the administration of the following baseline questionnaires to assess data unrelated to the medical examination:

- The pre‐exam section of the subjects acceptance questionnaire^20^ assesses subjects' confidence, concerns, and perceived utility of the WB‐MRI exam.
- The Big Five Inventory‐10^21^ is a self‐report questionnaire composed of 10 items that investigate the personological traits (i.e., agreeableness, conscientiousness, emotional stability, extroversion, and openness).
- Risk Perception questionnaire (modified version)^22^ examines the subjects' perception of illness severity/seriousness and personal risks. Perceived risk for cancer was assessed through two items in terms of absolute risk (“how likely or unlikely you think it is that you will develop cancer in the coming year”) and relative/comparative risk (“how likely do you think it is that you will develop cancer in the next year compared to other women/men of your age in Italy”).

Demographic data were also collected at this stage, in particular age, gender, educational level, and ethnicity. The educational level was calculated by the number of years required to attain the highest qualification.

After the WB‐MRI, two experienced radiologists with 15 and 10 years of expertise in WB‐MRI interpreted the exam and filled in the structured clinical report suggested in the ONCO‐RADS guidelines.^9^ The report included a statement regarding the individual's risk state, previous related examinations, and the imaging protocol adopted for the exam. Each AF was assigned to one of the seven anatomic regions (i.e., bones, head, neck, chest, abdomen, pelvis, and limbs) and classified as one of the five ONCO‐RADS categories. Finally, a summary statement was provided, describing the presence or the absence of any lesions suspicious of cancer and the related clinical recommendation.^9^

The participant received the clinical report through a brief meeting with the radiologist, who discussed the meaning of the findings and, if necessary, which examinations were required for further clinical investigation, ensuring that the patient well understood the outcome of the exam. Following the ONCO‐RADS guidelines, the individuals who reported AFs classified as category 2 were considered at low risk of cancer and were not suggested specific follow‐up. The individuals that reported AFs classified as ONCO‐RADS category 3 were considered at intermediate risk of cancer and performed further clinical examinations according to the anatomical region of the AFs. After this confrontation, a first assessment of the psychological health variables (T0) took place with an expert psychologist.

For the longitudinal evaluation of the psychological health variables, overall participants were re‐contacted by phone or email after around 1 year (T1) from the WB‐MRI exam. Finally, for 61 participants, it was also possible to obtain a further follow‐up after 4 years (T2) from the exam.

The following questionnaires were included in every psychological evaluation (see Data S1 for questionnaire scoring):

- The postexam section of the subjects acceptance questionnaire^20^ to assess the level of discomfort and satisfaction with the WB‐MRI exam.
- The short form‐12^23^ to investigate the subjects' QoL. It assesses the participant's perspective of the physical ability to function in terms of daily activities and bodily pain as well as mental, emotional, and social health (see Data S1).
- The Hamilton anxiety rating scale (HAM‐A^24^) to assess the level of anxiety. The HAM‐A consists of 14 items that measure both psychic symptoms (mental agitation and psychological distress) and somatic symptoms of anxiety (physical complaints related to anxiety).
- Patient health questionnaire^25^ to investigate depressive symptoms. It is a self‐report tool that analyzes the frequency of nine depressive symptoms (on a scale from “not at all” to “almost every day”) such as a lack of interest or satisfaction in daily activities, hopelessness, fatigue, or decreased appetite.

### Identification of AFs

We adopted a 5‐point rating scale, as reported in ONCO‐RADS guidelines,^9^ to classify each WB‐MRI finding in the clinical report, assessing the likelihood of being oncologically relevant (see Table 2). Specifically, ONCO‐RADS category 1 corresponds to “normal finding,” ONCO‐RADS category 2 corresponds to “benign finding highly likely,” ONCO‐RADS category 3 corresponds to “benign finding likely,” ONCO‐RADS category 4 corresponds to “malignant finding likely,” and ONCO‐RADS category 5 corresponds to “malignant finding highly likely.”^9^ For ONCO‐RADS categories 1 and 2, the individuals were considered at low risk of cancer and no specific follow‐up is required, whereas for ONCO‐RADS category 3 the individuals were considered at intermediate risk of cancer, and clarification of findings with specific imaging tests is required. For ONCO‐RADS categories 4 and 5, individuals were considered at high risk of cancer, and further investigations with or without histologic examinations are recommended.^9^

**TABLE 2:** ONCO‐RADS Category for Each AF

ONCO‐RADS Category	Clinical Classification
1	Normal
2	Benign finding highly likely
3	Benign finding likely
4	Malignant finding likely
5	Malignant finding highly likely

### Statistical Analyses

All statistical analyses were performed with SPSS (version 28; IBM Corp., Armonk, NY, USA). First, descriptives (frequency and percentage, mean, and standard deviation [SD]) were calculated. Second, Pearson's correlations between the key variables (i.e., personality dimensions, perceived risk of tumor, anxiety, depression, and QoL) at T0, T1, and T2 were tested. Finally, the general linear model (GLM) with repeated measures analysis of variance (ANOVA) was used to test possible changes in the psychological health outcomes (anxiety, depression, and QoL) over time, concerning the presence of AFs. For the statistical analysis, the AFs were categorized as a dichotomic variable: they were considered suspicious findings when belonging to ONCO‐RADS category ≥3, and not suspicious when they were classified as ONCO‐RADS categories 1 and 2. The AFs were considered both for the numerosity and for the presence of suspicious findings.

Considering that the study population could be a subpopulation of the general population, confounding socio‐demographic factors have been considered concerning personality traits to examine the influence of the responses to AFs. Perceived risk (cancer) and personality traits were inserted in the statistical analyses as covariates. The first two time points (T0 and T1) were considered in the main analyses.

Socio‐demographic variables were compared between the overall participants and the subgroup using a chi‐square test and two‐sample *t*‐test. Finally, additional analyses were conducted also considering T2. More specifically, to increase the strength of our conclusions, we performed the GLM repeated measures ANOVA for the subsample who participated in T2 (*n* = 61) considering the three time points (T0, T1, and T2). In case the assumption of sphericity was not met, the Greenhouse–Geisser correction was applied. A *P*‐value <0.05 was considered statistically significant.

## Results

### Participant Characteristics

A total of 121 participants were evaluated, 63% of them were male. The mean age was 52.61 years (SD = 11.39; range = 21–82 years). All participants were Italian and Caucasian. Regarding the educational level (mean years of education 16 ± 3.3 SD), 40 participants (33%) had a high school degree, while 81 participants (67%) had a university degree. Twenty‐three participants (19%) were smokers and nine participants (7.5%) were smokers in the past. Before the WB‐MRI exam, most of the participants (59%) did not present concerns about the procedure. However, 49 subjects (40%) reported a mild level of concern about the exam and only one participant showed a moderate level of concern. Regarding discomfort during WB‐MRI exams, most of the participants (44%) did not show any discomfort during the exam. However, 44 subjects (36%) reported a mild level of discomfort, 17 participants (14%) showed moderate discomfort, 6 participants (5%) reported a moderately severe level of discomfort, and one subject showed severe discomfort during the examination. After the WB‐MRI exams, 90% of patients reported high satisfaction with the exam. The remaining 10% of subjects showed moderate satisfaction regarding the procedure.

Compared with the entire sample, the subsample of 61 participants that were available for the 4‐year follow‐up exams did not statistically differ concerning the socio‐demographical data (i.e., age *P* = 0.639; education *P* = 0.579; gender *P* = 0.738).

### Abnormal Findings

Each of the 121 participants presented at least one AF according to WB‐MRI. The mean number of AFs in the sample was 4.21 (SD = 2.13; range = 1–11). Only 19 participants showed ONCO‐RADS category three findings, and the mean number was 0.18 (SD = 0.46; range = 0–3). None of the participants reported AFs identified with ONCO‐RADS categories 4 and 5.

Most AFs involved the bones (31.5%), with the narrowing of the spinal canal. Numerous AFs were also found in the abdomen (28%), pelvis (14.5%), chest (11.5%), and head (11.5%). With less frequency, AFs were also found in the neck (2.5%) and limbs (0.5%).

For the analysis, the number of AFs was codified into “low”^1^, ^2^, ^3^, ^4^ numbers (57.9%) or “high”^5^, ^6^, ^7^, ^8^, ^9^, ^10^, ^11^ numbers (42.1%) of AFs. Suspicious AFs were considered for absence (84.3%) or presence (15.7%).

### Psychological Health Outcomes

For each scale, results are reported in Table 3.

**TABLE 3:** Mean and SD of the Psychological Health Variables and Covariates

Variables	T0 (*n* = 121)	T1 (*n* = 121)	T2 (*n* = 61)
	Data M (SD)	Data M (SD)	Data M (SD)
Anxiety (0–56)	7.77 (7.04)	8.30 (7.27)	8.29 (7.60)
Depression (0–27)	3.26 (3.18)	3.35 (3.88)	4.52 (3.65)
Mental component QOL (0–100)	50.77 (9.01)	49.90 (9.96)	48.70 (9.21)
Physical component QOL (0–100)	52.86 (6.17)	52.58 (6.38)	53.87 (5.30)
BFI–agreeableness (1–5)	3.35 (0.98)	–	–
BFI–conscientiousness (1–5)	4.27 (0.78)	–	–
BFI–emotional stability (1–5)	3.40 (1.09)	–	–
BFI–extroversion (1–5)	3.18 (0.94)	–	–
BFI–openness (1–5)	3.87 (0.91)	–	–
Perceived risk–tumor (1–5)	3.08 (0.81)	–	–

QOL = quality of life; M = means; SD = standard deviation; BFI = Big Five Inventory.

Most participants reported low levels of anxiety at T0. Specifically, 74 participants (61%) had no/minimal anxiety, 33 subjects (28%) reported mild levels of anxiety, 10 participants (8%) showed moderate levels of anxiety, and 4 subjects (3%) presented severe levels of anxiety. Similarly, most participants showed low levels of depression. In particular, 87 participants (61%) had no/minimal depressive symptoms, 27 subjects (22%) reported mild levels of depression, 5 participants (4%) showed moderate levels of depression, and only 2 subjects (2%) presented moderately severe depressive symptoms.

Table 4 presents the correlations of the personality dimensions, perceived risk of tumor, and psychological health at T0. For the correlates of psychological health outcomes, anxiety showed a negative and significant correlation with emotional stability and extroversion [*r* = −0.33 and *r* = −0.24, respectively]. Moreover, depression was negatively correlated with emotional stability and conscientiousness [*r* = −0.28 and *r* = −0.24, respectively]., while it showed a positive significant correlation with perceived tumor risk [*r* = 0.29]. The mental component of QoL showed a positive and significant correlation with emotional stability and extroversion [*r* = 0.30 and *r* = 0.20, respectively].

**TABLE 4:** Correlations Between Personality Dimensions, Perceived Risk (Tumor), and Psychological Health Variables

Variables	1.	2.	3.	4.	5.	6.
1. BFI Agreeableness	–					
2. BFI Conscientiousness	0.01 *P* = 0.903	–				
3. BFI Emotional stability	−0.01 *P* = 0.950	0.14 *P* = 0.136	–			
4. BFI Extroversion	0.26 ***P* = 0.005**	0.15 *P* = 0.109	0.11 *P* = 0.253	–		
5. BFI Openness	0.05 *P* = 0.562	0.18 ***P* = 0.045**	−0.001 *P* = 0.994	0.07 *P* = 0.419	–	
6. Perceived risk (tumor)	0.03 *P* = 0.743	−0.14 *P* = 0.112	−0.12 *P* = 0.174	−0.05 *P* = 0.557	−0.26 ***P* = 0.004**	–
7. Anxiety	−0.02 *P* = 0.827	−0.14 *P* = 0.129	−0.33 ***P* < 0.001**	−0.24 ***P* = 0.009**	−0.01 *P* = 0.868	0.07 *P* = .0455
8. Depression	−0.04 *P* = 0.609	−0.28 ***P* = 0.002**	−0.24 ***P* = 0.008**	−0.16 *P* = 0.078	0.01 *P* = 0.873	0.29 ***P* = 0.001**
9. Mental component QOL	0.12 *P* = 0.204	0.13 *P* = 0.153	0.30 ***P* < 0.001**	0.20 ***P* = 0.025**	0.02 *P* = 0.801	−0.17 *P* = 0.058
10. Physical component QOL	−0.15 *P* = 0.101	0.07 *P* = 0.434	−0.02 *P* = 0.831	−0.03 *P* = 0.736	−0.06 *P* = 0.527	−0.05 *P* = 0.614

BFI = Big Five Inventory; QOL = quality of life.

A *p*‐value < 0.05 was considered statistically significant.

### Changes from T0, T1, and T2

Changes over time in psychological health variables are shown in Table 5. Concerning the psychological health outcomes at T1, participants showed a slight increase only in depressive scores from T0 to T1. Furthermore, the participants showed no significant differences in anxious and depressive symptoms according to the number of AFs ([*F*(1,112) = 0.11; *P* = 0.743] and [*F*(1,112) = 0.45; *P* = 0.503], respectively), nor for the presence of suspicious findings ([*F*(1,112) = 0.12; *P* = 0.729] and [*F*(1,112) = 0.42; *P* = 0.516], respectively). Similarly, regarding the mental component and physical component of QoL, no significant differences were found according to the number of AFs ([*F*(1,112) = 0.29; *P* = 0.593] and [*F*(1,112) = 0.30; *P* = 0.582]), nor for the presence of suspicious AFs ([*F*(1,112) < 0.01; *P* = 0.997] and [*F*(1,112) = 0.24; *P* = 0.626]).

**TABLE 5:** Changes from T0 to T1 in Psychological Variables, Concerning the Presence of AFs

	Anxiety	Depression	MCS QOL	PCS QOL
Time (T0‐T1)	*F* (1,112) = 3.00 *P* = 0.086 *η* ^2^ = 0.026	*F* (1,112) = 7.54 ***P* = 0.007** *η* ^2^ = 0.063	*F* (1,112) = 1.08 *P* = 0.300 *η* ^2^ = 0.010	*F* (1,112) = 0.00 *P* = 0.987 *η* ^2^ = 0.000
Time (T0‐T1) * agreeableness	*F* (1,112) = 0.55 *P* = 0.461 *η* ^2^ = 0.005	*F* (1,112) = 1.66 *P* = 0.200 *η* ^2^ = 0.015	*F* (1,112) = 0.00 *P* = 0.994 *η* ^2^ = 0.000	*F* (1,112) = 1.49 *P* = 0.225 *η* ^2^ = 0.013
Time (T0‐T1) * conscientiousness	*F* (1,112) = 0.94 *P* = 0.335 *η* ^2^ = 0.008	*F* (1,112) = 7.87 ***P* = 0.006** *η* ^2^ = 0.066	*F* (1,112) = 0.54 *P* = 0.462 *η* ^2^ = 0.005	*F* (1,112) = 0.00 *P* = 0.999 *η* ^2^ = 0.000
Time (T0‐T1) * emotional stability	*F* (1,112) = 0.58 *P* = 0.449 *η* ^2^ = 0.005	*F* (1,112) = 1.88 *P* = 0.173 *η* ^2^ = 0.017	*F* (1,112) = 0.16 *P* = 0.695 *η* ^2^ = 0.001	*F* (1,112) = 3.77 *P* = 0.055 *η* ^2^ = 0.033
Time (T0‐T1) * extroversion	*F* (1,112) = 1.62 *P* = 0.206 *η* ^2^ = 0.014	*F* (1,112) = 0.71 *P* = 0.400 *η* ^2^ = 0.006	*F* (1,112) = 0.05 *P* = 0.823 *η* ^2^ = 0.000	F (1,112) = 0.00 *P* = 0.963 *η*2 = 0.000
Time (T0‐T1) * openness	*F* (1,112) = 4.41 ***P* = 0.036** *η* ^2^ = 0.039	*F* (1,112) = 3.15 *P* = .079 *η* ^2^ = .027	*F* (1,112) = 2.62 *P* = 0.108 *η* ^2^ = 0.023	*F* (1,112) = 0.00 *P* = .956 *η* ^2^ = .000
Time (T0‐T1) * risk (tumor)	*F* (1,112) = 0.08 *P* = 0.779 *η* ^2^ = 0.002	*F* (1,112) = 0.71 *P* = 0.401 *η* ^2^ = 0.006	*F* (1,112) = 0.028 *P* = 0.868 *η* ^2^ = 0.000	*F* (1,112) = 0.24 *P* = 0.624 *η* ^2^ = 0.002
Time (T0‐T1) * suspicious findings (0–1)	*F* (1,112) = 0.12 *P* = 0.729 *η* ^2^ = 0.001	*F* (1,112) = 0.42 *P* = 0.516 *η* ^2^ = 0.004	*F* (1,112) = 0.00 *P* = 0.997 *η* ^2^ = 0.000	*F* (1,112) = 0.24 *P* = 0.626 *η* ^2^ = 0.002
Time (T0‐T1) * number of AFs (0–1)	*F* (1,112) = 0.11 *P* = 0.743 *η* ^2^ = 0.001	*F* (1,112) = 0.45 *P* = 0.503 *η* ^2^ = 0.004	*F* (1,112) = 0.29 *P* = 0.593 *η* ^2^ = 0.003	*F* (1,112) = 0.30 *P* = 0.582 *η* ^2^ = 0.003

In the first line, the dependent variable is reported.

MCS = Mental Health Component Summary Score; PCS=Physical Health Component Summary Score; QOL = quality of life; AFs = abnormal findings.

A *p*‐value < 0.05 was considered statistically significant.

The presence of suspicious AFs and the number of AFs were not significantly related to a change in psychological health outcomes [ranging from *P* = 0.503 to *P* = 0.997; for further details see Table 5]. Similarly, the different values in personality dimensions and perceived risk were not significantly associated with changes in the mental and physical components of QoL [ranging from *P* = 0.055 to *P* = 0.999; for further details see Table 5]. However, high levels of openness showed a role in the anxiety progression and high levels of conscientiousness showed a role in the depression progression.

Table 6 presents the results of the repeated measures ANOVA (*n* = 61). None of the results was significant, except for conscientiousness, which showed a significant interaction with depression over time, consistent with the results of the entire sample at T1 reported in the previous paragraph [*F* (1.708,90.544) = 3.40].

**TABLE 6:** Changes in Psychological Variables Over Time, Concerning the Presence of AFs

	Anxiety	Depression	MCS QOL	PCS QOL
Time (T0‐T1‐T2)	*F* (2,106) = 0.56 *P* = 0.571 *η* ^2^ = 0.011	*F* (1.708,90.544) = 2.77 *P* = 0.076 *η* ^2^ = 0.050	*F* (2,106) = 0.44 *P* = 0.644 *η* ^2^ = 0.008	*F* (2,106) = 1.81 *P* = 0.168 *η* ^2^ = 0.033
Time (T0‐T1‐T2) * Agreeableness	*F* (2,106) = 1.25 *P* = 0.290 *η* ^2^ = 0.023	*F* (1.708,90.544) = 2.15 *P* = 0.130 *η* ^2^ = 0.039	*F* (2,106) = 0.30 *P* = 0.744 *η* ^2^ = 0.006	*F* (2,106) = 0.38 *P* = 0.685 *η* ^2^ = 0.007
Time (T0‐T1‐T2) * conscientiousness	*F* (2,106) = 1.13 *P* = 0.326 *η* ^2^ = 0.021	*F* (1.708,90.544) = 3.40 ***P* = 0.045** *η* ^2^ = 0.060	*F* (2,106) = 1.67 *P* = 0.316 *η* ^2^ = 0.022	*F* (2,106) = 1.45 *P* = 0.239 *η* ^2^ = 0.027
Time (T0‐T1‐T2) * emotional stability	*F* (2,106) = 0.55 *P* = 0.576 *η* ^2^ = 0.010	*F* (1.708,90.544) = 0.67 *P* = 0.489 *η* ^2^ = 0.013	*F* (2,106) = 2.10 *P* = 0.127 *η* ^2^ = 0.038	*F* (2,106) = 1.35 *P* = 0.265 *η* ^2^ = 0.025
Time (T0‐T1‐T2) * Extroversion	*F* (2,106) = 1.21 *P* = 0.301 *η* ^2^ = 0.022	*F* (1.708,90.544) = 1.19 *P* = 0.164 *η* ^2^ = 0.034	*F* (2,106) = 0.78 *P* = 0.461 *η* ^2^ = 0.014	*F* (2,106) = 1.72 *P* = 0.184 *η* ^2^ = 0.031
Time (T0‐T1‐T2) * Openness	*F* (2,106) = 0.23 *P* = 0.792 *η* ^2^ = 0.004	*F* (1.708,90.544) = 0.50 *P* = 0.581 *η* ^2^ = 0.009	*F* (2,106) = 0.26 *P* = 0.771 *η* ^2^ = 0.005	*F* (2,106) = 0.16 *P* = 0.853 *η* ^2^ = 0.003
Time (T0‐T1‐T2) * Risk (tumor)	*F* (2,106) =0.98 *P* = 0.378 *η* ^2^ = 0.018	*F* (1.708,90.544) = 0.93 *P* = 0.385 *η* ^2^ = 0.017	*F* (2,106) = 2.72 *P* = 0.070 *η* ^2^ = 0.049	*F* (2,106) = 2.23 *P* = 0.112 *η* ^2^ = 0.040
Time (T0‐T1‐T2) * Suspicious findings (0–1)	*F* (2,106) = 0.97 *P* = 0.383 *η* ^2^ = 0.018	*F* (1.708,90.544) = 0.51 *P* = 0.574 *η* ^2^ = 0.010	*F* (2,106) = 0.35 *P* = 0.703 *η* ^2^ = 0.007	*F* (2,106) = 1.56 *P* = 0.215 *η* ^2^ = 0.029
Time (T0‐T1‐T2) * Number of AFs (0–1)	*F* (2,106) = 1.07 *P* = 0.348 *η* ^2^ = 0.020	*F* (1.708,90.544) = 0.44 *P* = 0.612 *η* ^2^ = 0.008	*F* (2,106) = 1.47 *P* = 0.234 *η* ^2^ = 0.027	*F* (2,106) = 1.29 *P* = 0.279 *η* ^2^ = 0.024

In the first line, the dependent variable is reported.

MCS = Mental Health Component Summary Score; PCS = Physical Health Component Summary Score; QOL = quality of life; AFs = abnormal findings.

A *p*‐value < 0.05 was considered statistically significant.

## Discussion

In this longitudinal study, we investigated the possible long‐term psychosocial consequences of the disclosure of AFs after WB‐MRI exams performed for cancer screening on asymptomatic subjects of the general population. Following the meeting with the radiologist and the explanation of the clinical report, most of the participants did not report high levels of anxiety despite the discovery of AFs. Indeed, only a small minority of subjects presented moderate to severe levels of anxiety. Similarly, most of the participants did not show the presence of relevant depressive symptoms. These findings are supported by recent studies, which have identified mild psychological distress in those patients who showed AFs following WB‐MRI exams.^14^, ^16^ Indeed, the presence of AFs had limited clinical relevance and therefore may cause moderate levels of concern. In addition, the subjects enrolled in the mentioned studies, as well as our sample, included asymptomatic individuals with a low pretest probability of severe clinical findings.^14^, ^16^

Although it is essential to analyze the psychological consequences of health events, other dispositional factors can influence the interpretation of the health news, allowing some individuals to perceive better health outcomes than others.^19^ In particular, personality traits play a crucial role in influencing individual responses to disease and in driving people's behavior in low‐risk health contexts (i.e., the discovery of non‐suspicious AFs).^19^ Moreover, a previous study found that personality traits have an impact on patients' acceptability and satisfaction with the WB‐MRI exam, underlining their influence on the patient's clinical experience.^11^ For these reasons, one main focus of our research was to investigate the role of personality traits in response to the disclosure of unexpected health outcomes.

The analysis of psychological health variables, personality traits, and risk perception reveals how certain features are interrelated following the disclosure of AFs through WB‐MRI exams. Our findings suggest that participants with low emotional stability, characterized by mood swings, susceptibility to negative emotions, and ease in interpreting ordinary events as threatening, tend to report high levels of anxiety and depressive symptomatology.^26^, ^28^ More precisely, the present sample showed that the level of anxiety was negatively correlated with the personality traits of emotional stability and extroversion. On the other hand, the level of depressive symptoms was positively correlated with the risk perception of having a tumor, and negatively correlated with the personality traits of conscientiousness and emotional stability. Indeed, subjects with higher procrastination, frequently associated with the personality trait of conscientiousness, reported mood deflection and lower psychological well‐being, which may enhance the risk of missing regular healthcare follow‐up exams.^27^, ^29^

Finally, the association between emotional stability (i.e., referred to the personality trait of neuroticism), anxiety, and depressive symptoms is supported by the presence of different studies conducted in both clinical and nonclinical samples,^30^, ^31^ and it could be partially explained by maladaptive emotion regulation strategies and rumination on the unexpected health outcomes of the WB‐MRI exams.^32^ Instead, participants with low extroversion, who are introverted, reserved, and less inclined to invest energy in social contexts^26^, ^27^ are prone to report a high level of anxiety. Specifically, in line with previous studies,^26^, ^33^ subjects with higher levels of extroversion and emotional stability reported a higher QoL, thus reflecting better emotional resilience, good stress management, higher assertiveness, and emotional expressiveness with higher related mental health functioning.^26^, ^27^

Along with psychological and personality traits characteristics, risk perception of having a tumor was also related to the interpretation of clinical outcomes. Subjects with a higher personal risk perception tend to report more depressive symptoms, lower QoL, and low openness. These findings may suggest that participants with an aversion to change experience more difficulties in implementing effective strategies to cope with the unexpected disclosure of AFs, showing psychological distress that has a detrimental effect on QoL.^26^, ^27^, ^34^

Other analyses were longitudinally conducted to assess the possible impact of the AFs disclosure after the WB‐MRI examination on mental health. In line with the results of Schmidt et al,^15^ our sample showed a significant increase in depressive symptoms after 1 year from the discovery of AFs. However, while Schmidt et al^15^ found moderate to severe psychological distress after the disclosure, in our sample the depressive symptoms remained on average within a mild clinical classification. This difference could be partially explained by the different modalities of communication of the clinical reports to the subjects. In the work of Schmidt et al,^15^ the participants received the communication of the clinical findings by a postal letter within 6 weeks of the WB‐MRI examination, while our sample received the clinical report directly from the radiologist, who ensured that participants completely understood the outcomes of the examination. Indeed, good communication between clinicians and patients can reduce emotional distress and increase satisfaction, facilitating participation in the consultation and increasing the patient's ability to cope with illness.^35^, ^36^

Concerning the 4‐year follow‐up examination, the increase in depressive symptoms in our subsample is no longer significant, thus potentially showing how, over time, the discovery of AFs does not affect the subject's emotional state. Our results seem to confirm that the discovery of AFs after the WB‐MRI exams has no lasting effects on QoL indicators, suggesting that the participants did not have to change their everyday activities in response to this clinical report, and did not statistically significantly influence the anxiety levels and depressive symptoms perceived by the subjects. This interpretation of the AFs by the subjects after a few years, as reported by other studies.^14^, ^16^ seems to promote the adoption of the WB‐MRI examination in clinical settings without psychosocial consequences on the participants.

Analyzing the role of personality traits in response to the disclosure of AFs over time, our data showed that conscientiousness seemed to be a risk factor for the development of depressive symptoms: subjects with more difficulty in pursuing long‐term goals and poor organization^26^, ^27^ tend to have a long‐term increase in depressive symptomatology.^26^, ^27^, ^36^ On the contrary, subjects with high openness (i.e., having an open mind and being good at making connections between different concepts) showed a greater vulnerability in experiencing anxiety after 1‐year follow‐up examinations. As a possible explanation of the last result, we might speculate that people processing more inputs may also experience more anxiety, especially when the obstacles concern the individual health status. In this regard, openness is strictly related to the concept of self‐awareness,^37^ an attitude characterized by paying attention to feelings and behaviors, which includes psychological rumination and self‐reflection.^38^, ^39^ In particular, subjects who showed high self‐awareness would be more inclined to identify goal‐related obstacles.^38^

Further research with a larger sample would be needed to clarify the relationship between the different personality traits and psychological health outcomes and better understand how personal characteristics may impact AFs perception following WB‐MRI exams. This field of research may contribute to extending the availability of the WB‐MRI and promoting the adoption of personalized medicine, defining communication strategies, encouraging participation in prevention programs, and improving the quality of services related to cancer screening for the general population.^40^ To achieve this objective, however, it is necessary to address the barriers that currently prevent the extension of the WB‐MRI to a larger portion of the population. The results from this study may be an incentive to overcome those existing barriers, highlighting the benefits that the use of WB‐MRI can bring within clinical practice and to patients themselves.

### Limitations

Given the lack of a control group, our study represents an observation of a particular sample of asymptomatic subjects with the detection of AFs after their WB‐MRI exams. Despite further studies are needed, these preliminary results might be relevant for the understanding of the psychosocial impact of undergoing WB‐MRI exams. Furthermore, given the importance of the psychological interpretation provided by the participants, we chose to adopt several self‐report tools to identify the personal experience of the clinical examination. Although the instruments used are validated and commonly used, the self‐report questionnaires are more prone to personal bias. Finally, another limitation concerns the small sample size of the long‐term follow‐up group. The enrollment of participants began in 2019 and reached a considerable number of subjects; however, the pandemic of COVID‐19 of the last years made it more difficult to contact participants during the specific timeframe, leading to an inevitable drop‐out rate.

## Conclusion

Our results, obtained by longitudinal monitoring, may suggest that WB‐MRI exams can be used in asymptomatic subjects of the general population without negative long‐term psychosocial consequences. Despite the discovery of AFs including even highly suspicious AFs, participants may not show changes in the QoL indicators, level of anxiety, and depressive symptoms perceived after a 4‐year follow‐up interval. Instead, we found a slight increase in depressive symptoms after 1 year from the disclosure of AFs following the WB‐MRI exams. This may suggest a short‐term effect on psychosocial distress. Finally, certain personality traits contribute to the psychological distress experienced by individuals with AFs after WB‐MRI exams.

## Author Contributions

Lorenzo Conti: conceptualization, investigation, formal analysis, methodology, writing–original draft, and writing–review and editing. Davide Mazzoni: conceptualization, data curation, methodology, and writing–review and editing. Chiara Marzorati: data curation, and writing–review and editing. Roberto Grasso: writing–review and editing. Giuseppe Petralia: funding acquisition, project administration, and supervision. Gabriella Pravettoni: funding acquisition, project administration, and supervision. All authors read and approved the final manuscript.

## Funding Information

This work was partially supported by Fondation IEO‐CCM. The present work was partially supported by the Italian Ministry of Health with Ricerca Corrente and 5 × 1000 funds for the European Institute of Oncology IRCSS.

## Supporting information


**Data S1.** Supporting Information.
