# Supplementary material for: Observations Regarding the Detection of Abnormal Findings Following a Cancer Screening Whole‐Body MRI in Asymptomatic Subjects: The Psychological Consequences and the Role of Personality Traits Over Time
Source: J Magn Reson Imaging. 2024 May 31;61(2):634–45. doi: 10.1002/jmri.29461 (PMC11706315; doi:10.1002/jmri.29461)
Supplement: Supplementary file 1 — Data S1. Supporting Information. [file JMRI-61-634-s001.docx]

**Supplementary material**

*Clinical assessment*

The clinical evaluation of anxiety (HAM-A) and depression (PHQ-9) considered the literature cut-offs and divided the severity of symptoms as follows: absence or minimal, mild levels, moderate and severe levels (1, 2). The score of SF-12 was divided into the Mental Health Component Summary Score (MCS), which assesses social functioning and general mental health, and the Physical Health Component Summary Score (PCS) which evaluates general physical health and functioning (3).

*Results*

Supplementary Table 1 presents the correlations of the psychological health variables at each time point investigated (e.g. T0, T1, and T2). Analysis showed how the levels of anxiety and depression are associated with each other during all the time points with moderate to strong correlations. In addition, both psychological variables are negatively correlated with the quality of life variables, especially for the mental component which showed moderate to strong correlation during the three-time point.

**Supplementary table 1.** Correlations between the health variables at T0, T1, and T2.

|  | 1. | 2. | 3. | 4. | 5. | 6. | 7. | 8. | 9. | 10. | 11. |
| --- | --- | --- | --- | --- | --- | --- | --- | --- | --- | --- | --- |
| **T0** | | | | | | | | | | | |
| 1. ANX | - |  |  |  |  |  |  |  |  |  |  |
| 2. DEP | .47  **p<.001** | - |  |  |  |  |  |  |  |  |  |
| 3. MCS QOL | -.42  **p<.001** | -.51  **p<.001** | - |  |  |  |  |  |  |  |  |
| 4. PCS QOL | -.26  **p=.003** | -.18  p=.052 | -.22  **p=.015** | - |  |  |  |  |  |  |  |
| **T1** | | | | | | | | | | | |
| 5. ANX | .33  p<.001 | .16  p=.084 | -.13  p=.157 | -.12  p=.174 | - |  |  |  |  |  |  |
| 6. DEP | .25  **p=.006** | .37  **p<.001** | -.21  **p=.024** | -.07  p=.474 | .66  **p<.001** | - |  |  |  |  |  |
| 7. MCS QOL | -.18  **p=.042** | -.23  **p=.011** | .31  **p<.001** | .00  p=.973 | -.64  **p<.001** | -.61  **p<.001** | - |  |  |  |  |
| 8. PCS QOL | -.21  **p=.023** | .02  p=.815 | -.03  p=.761 | .54  **p<.001** | -.24  **p=.009** | -.25  **p=.006** | -.10  p=.292 | - |  |  |  |
| **T2** | | | | | | | | | | | |
| 9. ANX | .32  **p=.011** | .14  p=.285 | -.15  p=.239 | -.26  **p=.043** | .49  **p<.001** | .38  **p=.002** | -.35  **p=.005** | -.12  p=.342 | - |  |  |
| 10. DEP | .35  **p=.005** | .19  p=.133 | -.18  p=.154 | -.32  **p=.011** | .41  **p=.001** | .45  **p<.001** | -.42  **p<.001** | -.15  p=.232 | .76  **p<.001** | - |  |
| 11. MCS QOL | -.26*  **p=.044** | -.19  p=.138 | .20  p=.120 | .15  p=.243 | -.25  p=.050 | -.29  **p=.024** | .49  **p<.001** | -.04  p=.736 | -.52  **p<.001** | -.75  **p<-.001** | - |
| 12. PCS QOL | -.13  p=.321 | .15  p=.239 | -.15  p=.246 | .48  **p<.001** | -.22  p=.087 | -.03  p=.800 | -.10  p=.425 | .58  **p<.001** | -.34  **p=.007** | -.21  p=.101 | -.13  p=.311 |

ANX=Anxiety; DEP=Depression; MCS=Mental Health Component Summary Score; PCS=Physical Health Component Summary Score; QOL=Quality of Life.

**Supplementary Table 2** WB-MRI scanning protocol

| **Scanning Parameters (1.5T)** | **Chest / Abdomen / Pelvis** | | | **Spine** | |
| --- | --- | --- | --- | --- | --- |
| **Image Contrast** | T1-Dixon | T2 | DWI | T1 | T2 STIR |
| **Imaging Sequence** | GRE | HASTE | SSH SE EPI | TSE | TSE |
| **Orientation** | Axial | Axial | axial | Sagittal | Sagittal |
| **Echo / Repetition Time (ms)** | 2.39,4.77 / 6.65 | 74 / 800 | 62 / 6550 | 9.3 / 350 | 60 / 2560 |
| **Field of view (mm)** | 430 | 430 | 430 | 400 | 400 |
| **Matrix** | 352 x 209 | 320 x 175 | 132 x 120 | 448 x 224 | 320 x 160 |
| **Slices per Station / # Stations** | 72 / 4 | 191 / 1 | 50 / 4 | 16 / 2 | 16 / 2 |
| **Flip angle (°)** | 20.5 | 90 | 90 | 90 | 90 |
| **Slice Thickness / Gap (mm)** | 3.5 / 0 | 5 /1 | 5 / 0 | 4 / 0.4 | 4 / 0.4 |
| **Fat Suppression** | - | - | STIR | - | STIR |
| **Respiratory Control** | Breath-Hold | Breath-Hold |  |  | - |
| **b-values (s/mm2)** | - | - | 50, 900 | - | - |
| **Acquisition Time (min:sec)** | 1:04 | 2:46 | 15:04 | 3:32 | 3:10 |

GRE=Gradient-echo; HASTE=Half-Fourier Acquisition Single-shot Turbo-spin Echo; TSE=Turbo-Spin Echo; SSH SE EPI= Single-Shot Spin-Echo Echo Planar Imaging; STIR=Short Tau Inversion Recovery.

**References**

1. Matza LS, Morlock R, Sexton C, Malley K, Feltner D: Identifying HAM-A cutoffs for mild, moderate, and severe generalized anxiety disorder. *Int J Methods Psychiatr Res* 2010; 19:223–232.
2. Kroenke K, Spitzer RL, Williams JBW: *The PHQ-9 Validity of a Brief Depression Severity Measure*. 2001.
3. Ottoboni G, Cherici A, Marzocchi M, Chattat R: Algoritimi di calcolo per gli indici PCS e MSC del questinario sf-12. 2010.
